# An Overview of Adaptive Designs and Some of Their Challenges, Benefits, and Innovative Applications

**DOI:** 10.2196/44171

**Published:** 2023-10-16

**Authors:** Hongjian Zhu, Weng Kee Wong

**Affiliations:** 1 Statistical Innovation Group, AbbVie Inc. Virtual Office Sugar Land, TX United States; 2 Department of Biostatistics Fielding School of Public Health University of California at Los Angeles Los Angeles, CA United States

**Keywords:** doubly adaptive biased coin designs, model-based optimal designs, particle swarm optimization, repair mechanism

## Abstract

Adaptive designs are increasingly developed and used to improve all phases of clinical trials and in biomedical studies in various ways to address different statistical issues. We first present an overview of adaptive designs and note their numerous advantages over traditional clinical trials. In particular, we provide a concrete demonstration that shows how recent adaptive design strategies can further improve an adaptive trial implemented 13 years ago. Despite their usefulness, adaptive designs are still not widely implemented in clinical trials. We offer a few possible reasons and propose some ways to use them more broadly in practice, which include greater availability of software tools and interactive websites to generate optimal adaptive trials freely and effectively, including the use of metaheuristics to facilitate the search for an efficient trial design. To this end, we present several web-based tools for finding various adaptive and nonadaptive optimal designs and discuss nature-inspired metaheuristics. Metaheuristics are assumptions-free general purpose optimization algorithms widely used in computer science and engineering to tackle all kinds of challenging optimization problems, and their use in designing clinical trials is just emerging. We describe a few recent such applications and some of their capabilities for designing various complex trials. Particle swarm optimization is an exemplary nature-inspired algorithm, and similar to others, it has a simple definition but many moving parts, making it hard to study its properties analytically. We investigated one of its hitherto unstudied issues on how to bring back out-of-range candidates during the search for the optimum of the search domain and show that different strategies can impact the success and time of the search. We conclude with a few caveats on the use of metaheuristics for a successful search.

## Introduction

There is active research on adaptive designs and their many applications in different areas of research. In particular, many types of adaptive designs and their variants have been proposed in the literature for different purposes in clinical trials. Adaptive designs are highly flexible and can address a multitude of important issues in clinical trials. They use accumulating data to modify various aspects of the study design to address emerging statistical problems in an ongoing trial in a preplanned manner. The planned interim data analysis can monitor the progress of the trial and can use various strategies to preempt having an underpowered study, covariate imbalance, or ethical concerns. For example, in the simplest case, when there are 2 treatment groups to be compared and both treatments are equally effective or equally ineffective, a lot of cost and labor could have been saved if the trial is adaptively designed for early termination and patients are not put in trials needlessly. Jennison and Turnbull [1] present the fundamentals of design and analysis of various adaptive trials, including details on the flexibility to stop the trial during interim looks either for efficacy or futility to decrease the original sample size for ethical and cost reasons. In particular, the seamless phase 2/3 designs allow treatment or dose selection at an interim analysis and use data from both phases to conduct a final comparative evaluation of efficacy with a single protocol [2]. Another monograph on sequential experimentation in clinical trials is the study by Bartroff et al [3].

Chow and Chang [4] summarized adaptive designs into the following types: adaptive randomization, group sequential designs, sample size re-estimation designs, drop-the-loser designs, adaptive dose-finding designs, biomarker-adaptive designs, adaptive treatment-switching designs, hypothesis-adaptive designs, adaptive seamless phase 2/3 trial designs (ASDs), and multiple adaptive designs. Rosenberger and Lachin [5] distinguished the randomization procedures into 4 classes: complete randomization, restricted randomization, covariate-adaptive randomization, and response-adaptive randomization (RAR). Complete randomization is unbiased coin tossing. Restricted randomization is used to balance the treatment assignments. Covariate-adaptive randomization is used to ensure the balance between treatment arms regarding specific known covariates.

RAR is used to address ethical concerns, such as allocating patients to more effective treatment groups, making inferences more efficiently, and saving costs. Interestingly, despite the large number of publications in adaptive methodology, many clinical trials are not adaptively designed. We offer some possible reasons: (1) many clinical trialists may not be familiar with the many benefits of modern adaptive approaches; (2) there is very limited software for implementing and analyzing adaptive designs, and when they are available, they can be prohibitively expensive; (3) compared with nonadaptive trials, the statistical planning for adaptive trials requires more technical work; (4) their administration is also more time-consuming because a participant or participants have to be randomized carefully and monitored throughout the trial; and (5) people tend to use well-established design methods and have concerns whether the regulatory agencies will accept results from an innovative adaptive design. Finally, and more importantly, there is no gold standard for adequately and fairly comparing the adaptive strategies. The problem is compounded by a plethora of new adaptive designs being proposed frequently, and they are invariably supported by simulations in a restricted setting only. Trial integrity, which includes strict adherence to all aspects of the protocol, and implementation complexity can still add time and cost to running an adaptive trial, despite fewer patients being required. We have proposed several ways to facilitate the broader use of adaptive designs in practice. They include promoting greater awareness of the capabilities of various adaptive designs over traditional designs with concrete illustrations using easy-to-use and free, modern web-based tools for finding optimal adaptive designs for different clinical settings. To this end, this paper has 4 aims. The first is to provide an overview of adaptive designs, and the second aim is to demonstrate the usefulness of recent adaptive strategies using the notable Indacaterol to Help Achieve New Chronic Obstructive Pulmonary Disease Treatment Excellence (INHANCE) trial [6] as an example. Third, we present several interactive websites for finding optimal designs, adaptive or not. Presently, they are scattered throughout the literature, and collecting some of them in one place in the internet era can be helpful to promote interest in adaptive designs and facilitate practitioners to find efficient clinical trial designs after a few strokes on the keyboard. The fourth purpose of this paper is to introduce nature-inspired metaheuristic algorithms to solve increasingly challenging design problems and recognize their emerging applications in designing biomedical studies. Particle swarm optimization (PSO) is an exemplary algorithm, and we have described some of its recent applications in the design of clinical trials. Similar to other algorithms, it has intriguing properties, and we investigated one of its hitherto unstudied, interesting features.

Adaptive Designs section briefly reviews selected adaptive design methods, and the following Section demonstrates how recent adaptive design techniques can be used to further improve the adaptive INHANCE trial [6] conducted approximately 13 years ago. Section 4 (Web-based tools) discusses interactive websites with various abilities to find various types of trial designs. Section 5 (Metaheuristics) presents an overview of nature-inspired metaheuristic algorithms, which are a modern class of assumptions-free general purpose optimization tools. We focused on one such exemplary algorithm called PSO and some of its recent applications to design studies for biomedical studies. In addition, we investigated, for the first time, one aspect of its many moving parts and have shown when search particles for the optimum move beyond the search domain, how they are brought back into the search domain can be consequential. The last section concludes with a summary of the benefits of adaptive designs and metaheuristics along with commentaries on their cautionary use in practice.

### Adaptive Designs

There are many types of adaptive designs, and we reviewed only a few in this paper. The selected ones are either commonly used or viewed as being more cutting-edge.

#### Sequential Multiple Assignment Randomized Trial Design

Diseases such as HIV and cancer require a series of treatments over time. We call such a treatment sequence a dynamic treatment regime (DTR). These DTRs are often tailored to an individual’s treatment and disease history, their response to treatments, and the patient’s characteristics.

There are 3 components for a DTR. The first is the treatment options. The second is the critical decision stage when future treatments are decided based on earlier results. The third component is to tailor variables that can be used to develop personalized medicine.

The Sequential Multiple Assignment Randomized Trial (SMART) design [7] that follows specific DTRs to randomize individuals multiple times has been proposed to develop the DTR and detect the treatment effects of these DTRs. A typical SMART design consists of 2 stages. In the first stage, individuals are randomized to different treatments. On the basis of the responses and patient characteristics, these individuals are subsequently randomized to the treatment options in the second stage. If the disease or the treatment has a long assessment period, the SMART design and other adaptive designs are likely going to be inappropriate.

A special feature of the SMART design is that it adapts the individual’s future treatment based on the individual’s previous responses. This is different from other adaptive designs, where treatment assignment depends on the cumulative results from other patients.

#### Adaptive Seamless Phase 2/3 Clinical Trial Designs

Traditionally, clinical trials are conducted in a sequential manner, with phase 1 trials evaluating the safety, phase 2 trials determining efficacy and identifying the optimal dose, and phase 3 trials confirming the efficacy and assessing safety in larger populations. This approach has proven to be successful in identifying new treatments for a wide range of diseases. However, sequential trials can be lengthy and expensive, and they may not provide the most efficient means of evaluating new interventions.

Combining the different phases of a clinical trial seamlessly into one trial is one way to quicken the process. For example, they may be a seamless phase 1/2 trial or a seamless phase 2/3 trial. Since the US Food and Drug Administration (FDA) has promoted streamlining clinical trials [8], it has been desirable to develop new treatments quickly without compromising the integrity and validity of the development process. To this end, the FDA drafted guidelines to encourage seamless clinical trials [9]. Recently, Project Optimus was initiated by the FDA Oncology Center of Excellence to transform the current approach to dose selection for cancer treatments in the field of oncology, which will surely bring a new wave of research on ASD. With ASD, a single protocol for the entire seamless clinical trial is sufficient, which eliminates the lag time between the 2 phases and can save ≥6 months. This approach also avoids the need to design separate trials for each experimental treatment and enables the use of data from both phases in the final analysis, resulting in a significant reduction in sample size. The upshot is tremendous savings in cost and time for pharmaceutical companies.

A typical seamless phase 2/3 clinical trial compares multiple experimental treatments or drug doses with a control in the phase 2 trial, and the best candidates based on certain criteria will be chosen to enter the phase 3 trial [2]. The data analysis and hypothesis testing are based on data from both the phases. Because we choose the best in the middle of the procedure and there are multiplicity issues, it is important to control the type 1 error rate. We typically use the closure principle [10] with the combination test [11] and multiple testing approaches, such as the Simes test [12] and Dunnett test [13] to control the familywise type 1 error rate.

A seamless clinical trial requires a protocol for the whole procedure, resulting in no lead time between the 2 phases and often saving ≥6 months of trial time. In addition, we do not have to design different trials for each experimental treatment and can use data from both phases for the final data analysis, thereby reducing the sample size noticeably. The upshot is that pharmaceutical companies can save costs considerably.

Ma et al [14] studied seamless phase 2/3 clinical trials with covariate-adaptive randomization and provided a theoretical foundation for the complex procedure. On the basis of their work, a simple modified version of the *t* test can be used, and the power of detecting the treatment effect can be increased by approximately 10% compared with the traditional approach.

#### Group Sequential Designs

A group sequential trial determines whether the trial should be terminated early based on interim evidence of efficacy, harm, or futility while preserving statistical error rates. It is appealing based on ethical, administrative, and economic considerations [1]. A group sequential trial can detect unsafe treatment regimens earlier and take action as soon as possible (ethical); it can ensure that the experiment is executed as required in the protocol (administrative); and it can stop the trial earlier so that fewer patients are required and there are savings in terms of time and money (economic).

A group sequential study typically consists of *K* analyses and hypothesis testing. Thus, we have presented a sequence of *K* test statistics. Following a unified approach, we can construct such test statistics for various types of responses for which these statistics share a common joint canonical distribution. Furthermore, by a simple transformation, these sequential statistics will follow the Brownian motion. In the literature, various approaches have been proposed to control the type 1 error rate for Brownian motion. Subsequently, the validity of the group sequential design is guaranteed.

Zhu and Hu [15,16] studied the sequential monitoring of 2 families of RAR designs, doubly adaptive biased coin design (DBCD) and urn models. Zhu and Hu [17] investigated the sequential monitoring of covariate-adaptive randomized clinical trials. These studies successfully combined the adaptive randomization procedures and adaptive analysis approaches in one clinical trial and achieved various design objectives.

As expected, adaptive strategies are motivated differently and have their own unique properties. Thus, the user should carefully select an appropriate adaptive design with the main objective or objectives of the trial in mind. Table 1 lists some of the advantages and disadvantages of the 3 adaptive strategies described.

**Table 1:** Advantages and disadvantages of 3 adaptive clinical trial designs in Section 2.

Design	Advantages	Disadvantages
SMART^a^ design	Allows for multiple interventions To be tested within 1 trial Provides the ability to adapt treatment based on patient response and lead to a more personalized approach to treatment	Can be complex and require more resources to implement May require a longer trial period May result in more dropouts owing to multiple intervention changes Requires careful planning to ensure statistical validity Can be more challenging to analyze and interpret results
ASD^b^	Allows for the use of phase 2 data in the final data analysis for filing Allows for the possibility of early termination, especially for futility Can reduce the overall trial duration, cost, and sample size Can compare multiple treatment arms and drop inferior ones early	Requires careful planning to ensure statistical validity Modeling the relationship between short and long end points in trials with time-to-events end points might be challenging The type 1 error rate control requires careful design and planning The testing approaches may not be the most powerful ones May result in more changes to the trial protocol Can be more challenging to analyze and interpret results
GSD^c^	Allows for early termination of the trial for superiority and futility Allows for early termination for safety issues Allows for early termination for protocol violation Can reduce the trial duration Can reduce the cost Can reduce sample size Can allow for comparison of multiple treatment arms	Requires careful planning to ensure statistical validity May require a larger maximum sample size to achieve statistical power May result in more changes to the trial protocol Can be more challenging to analyze and interpret results

^a^SMART: Sequential Multiple Assignment Randomized Trial.

^b^ASD: adaptive seamless phase 2/3 trial design.

^c^GSD: group sequential design.

### How Adaptive Strategies Can Improve Clinical Trials

#### Overview

The INHANCE trial [6] is a double-blinded, adaptive seamless phase 2/3 clinical trial of inhaled indacaterol to treat chronic obstructive pulmonary disease that is a chronic lung inflammation disease leading to poor airflow from the lungs and long-term breathing problems. The INHANCE trial implements *stratification randomization* to randomly allocate patients to ensure a balance in smoking status. In the dose-finding stage, patients were enrolled and randomly allocated to 7 treatment arms containing 4 doses of indacaterol, a placebo, and 2 active controls (formoterol and tiotropium). After 770 patients had completed 2 weeks of treatment, an interim analysis was performed by an independent statistician, and 2 indacaterol doses, the placebo and tiotropium, were selected by an independent data monitoring committee based on unblinded data.

The criteria for choosing treatment arms include efficacy, early bronchodilator effect, and safety. Finally, in the efficacy confirmation stage, 1683 patients were randomly allocated to the chosen treatment arms. With stratification randomization, the concern about the apparent confounding factor, smoking status, was addressed, and the results are more persuasive. With the adaptive seamless design, the data from the 2 stages can be used for the final analysis, leading to fewer required patients and a shorter overall duration compared with the traditional separate phase 2/3 trials. This means that such a trial saves costs and is efficient and ethical. This trial has significantly promoted the development of adaptive seamless phase 2/3 clinical trials and has received attention from the *New England Journal of Medicine* [18].

#### Example: An Enhanced Design for the INHANCE Trial

In the INHANCE trial, stratification randomization was used in the seamless phase 2/3 clinical trials to ensure balance across specific covariates. The advantage of stratified randomization in improving the efficiency of estimators and power of tests is more evident in a small trial (even with stratified analysis) than in large trials, but such an advantage will fade away as the difference in the size of strata becomes larger [19]. Although equal allocation at the outset of a trial may be appropriate in light of a clinical equipoise principle, it may become unethical if accumulating data suggest that one treatment is inferior. To address this ethical concern, RAR can be used, which sequentially assigns patients based on their previous treatment assignments and responses. This approach can alleviate the issue and offers appealing features. Here, we proposed an adaptive seamless trial design with a type of RAR to further achieve ethical objectives by assigning more patients to better treatments and reducing the number of failures.

The concept of RAR can be traced back to the Thompson [20] proposition that patients should be randomized to a treatment based on the probability that the treatment would be the most effective. Subsequently, RAR evolved into 2 families of frequentist methodologies, namely urn models and DBCDs [21].

For space consideration, we focused on the DBCD [21] that can target any theoretically optimal allocation proportions based on certain optimal criteria. Specifically, we first formulated the main objectives mathematically to reflect the trial objectives that could be maximizing the power for a fixed sample size or minimizing the total number of failures for a preselected level of power of a test. We derived the theoretically optimal allocation proportion that is a function of unknown parameters to achieve these objectives. Using the DBCD, we sequentially estimated these unknown parameters and updated the estimated targeted allocation proportions for each patient. Finally, we used the DBCD’s allocation probability function that considers both the estimated optimal allocation proportion and the actual allocation proportion to assign the next patient.

In this application, we used a simpler version of the DBCD by only using the estimated targeted allocation proportion to demonstrate its application to improve ethical benefits. We assumed that the outcome is binary and the trial has 3 experimental treatment arms and 1 control arm. The proposed step-by-step design procedure is as follows:

1. In the first stage, *m_0_* patients are assigned to each of the 4 treatments by fixed design to obtain initial parameter estimates. When the *m*th (*m*>4*m_0_*) patient enters the trial, calculate 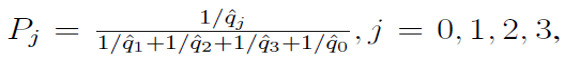, where 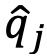 is the estimated failure rate of treatment *j* based on all the previous responses and treatment assignments.
2. Assign the *m*th patient to treatment *j* with probability *P_j_*. This RAR design will assign more patients to the better-performing treatments.
3. At the end of the first stage, choose the best-performing treatment arm (eg, treatment *M*) and the control arm to enter the second stage.
4. In the second stage, assign each new patient to treatment *j* with probability 
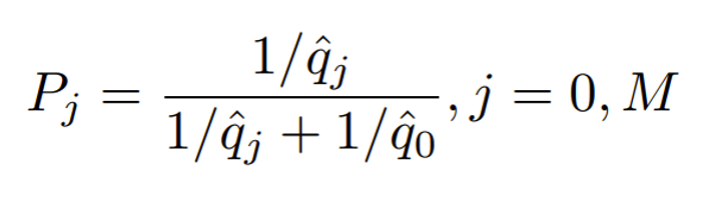
.

At the end of the trial, we tested

H_0_: *p_M_* = *p*_0_ versus H_1_: *p_M_ > p*_0_

using the closure principle [10] combined with the inverse chi-square method [22] and the Simes test [12].

We conducted a numerical study to evaluate this design strategy. We used 10,000 replications and assumed that there are 200 patients in the first stage and 300 patients in the second stage. Patients enter the trial sequentially and are randomly assigned to the treatment groups using the abovementioned randomization procedure. Table 2 compares the performance of our method with complete randomization (CompR). We found that, under the null hypothesis, our method can effectively control the type 1 error rate (α). We reported 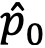 to demonstrate the accuracy and precision of the parameter estimators following our method. We also demonstrated the ethical advantages of our design by reporting the actual allocation proportion to the control group (ρ_0_) and the total number of failures (failure). The SDs are shown in parentheses. We observed that our method is ethically more advantageous than the traditional design by assigning approximately 7% more patients to the treatment arms instead of the control arm and reducing up to 7 failures while keeping the power at the same level as complete randomization under the alternative hypothesis.

In conclusion, the incorporation of innovative adaptive randomization into the novel adaptive seamless design has enabled us to achieve the ethical objective of assigning more patients to the better treatment arm, resulting in fewer failures. This achievement was accomplished without sacrificing the efficient objective and the original advantages of the adaptive seamless design. In addition, the adaptive randomization design has been found to aid patient enrollment and accelerate clinical trials, as noted by Tehranisa and Meurer [23]. This is particularly important in rare disease trials and for pharmaceutical companies seeking to secure funding for new trials.

**Table 2:** Performance of the proposed design procedure for an adaptive phase 2/3 clinical trial design with response adaptive randomization with 3 treatments and a placebo group relative to that in a complete randomization (CompR) procedure.^a^.

(*p*_1_*,* *p*_2_*,* *p*_3_*,* *p*_0_)	Design	Type 1 error rate or power	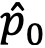 (SD)	Proportion, ρ_0_ (SD)	Failure, n (SD)
(0.4*,* 0.4*,* 0.4*,* 0.4)	DBCD^b^	.023	0.399 (0.034)	0.400 (0.018)	300 (11)
(0.4*,* 0.4*,* 0.4*,* 0.4)	CompR	.026	0.400 (0.035)	0.400 (0.021)	300 (11)
(0.5*,* 0.5*,* 0.5*,* 0.5)	DBCD	.025	0.499 (0.036)	0.400 (0.021)	250 (11)
(0.5*,* 0.5*,* 0.5*,* 0.5)	CompR	.025	0.500 (0.035)	0.400 (0.021)	250 (11)
(0.6*,* 0.6*,* 0.6*,* 0.6)	DBCD	.025	0.598 (0.035)	0.400 (0.024)	200 (11)
(0.6*,* 0.6*,* 0.6*,* 0.6)	CompR	.023	0.600 (0.035)	0.400 (0.021)	200 (11)
(0.45*,* 0.55*,* 0.6*,* 0.4)	DBCD	.91	0.400 (0.038)	0.345 (0.023)	247 (13)
(0.45*,* 0.55*,* 0.6*,* 0.4)	CompR	.90	0.400 (0.035)	0.400 (0.021)	253 (12)
(0.6*,* 0.65*,* 0.7*,* 0.5)	DBCD	.91	0.499 (0.039)	0.332 (0.027)	194 (13)
(0.6*,* 0.65*,* 0.7*,* 0.5)	CompR	.91	0.500 (0.035)	0.400 (0.021)	201 (12)
(0.65*,* 0.65*,* 0.7*,* 0.5)	DBCD	.93	0.499 (0.039)	0.332 (0.026)	192 (12)
(0.65*,* 0.65*,* 0.7*,* 0.5)	CompR	.93	0.500 (0.035)	0.400 (0.021)	199 (11)

^a^The simulated results in the first 6 rows are obtained under the null hypothesis using a significance level of 0.025. The remaining rows are simulated results obtained under the alternative hypothesis, along with the power attained. The ρ_0_'s are the proportions of patients assigned to the control group.

^b^DBCD: doubly adaptive biased coin design.

### Web-Based Tools

In the internet age, it is helpful to have interactive websites enabled with programs that can quickly generate the results the user is looking for after they provide input to the problem of their interest. Although this is common in engineering and computer science, it is less common in biostatistical and statistical research areas. We reviewed some areas in this paper by describing some websites and their capabilities and limitations.

A most remarkable website that seems to contain the most codes for generating many optimal designs and analyzing various types of clinical trials is housed at the Biostatistics Department at MD Anderson [24]. There are more than 70 programs available for free download. It has nearly 25,000 visitors to date, which makes it probably the most visited site for biomedical researchers interested in finding efficient designs and performing various analyses for phase 1, phase 2, and phase 3 clinical trials. The analysis methods include both frequentist and Bayesian approaches; an example of the latter is one that uses the Bayesian chi-square test to evaluate the goodness of fit for 7 common models for right-censored time-to-event data. Both the design and analysis methods are available for clinical trials with a single arm, 2 arms, or multiple arms, including many codes for performing various adaptive randomization schemes. Some of the programs are aimed at pedagogy; for example, there are codes that show how the beta-binominal densities vary when the parameters are changed or sample size calculations for various common situations.

Another useful site, albeit on a smaller scale, is Professor Ivanova’s home page [25] in the Department of Biostatistics at the University of North Carolina at Chapel Hill [26]. It is one of a few interactive websites, where the visitor inputs the design parameters and the site returns the desired design. For instance, the celebrated Simon 2-stage designs for phase trials [27] are displayed after one inputs the type 1 and 2 error rates and the values of the binary response rates under the null and alternative hypotheses. The site also allows the user to generate other types of designs useful for phase 1 and 2 trials, and they include (1) the 2-stage design by Fleming [28]; (2) a Simon-like design with relaxed futility stopping [29]; (3) a 2-stage design for ordinal outcomes; (4) rapid enrollment design for phase 1 trials; and (5) continuous monitoring for toxicity using a Pocock-type boundary.

However, there are other web-based tools scattered elsewhere with limited capacity for finding a few types of optimal designs. For instance, in the study by Collins et al [30], there is a web link [31] that finds locally *D*-optimal designs or Bayesian nonadaptive *D*-optimal designs for estimating parameters or a user-selected percentile in a beta regression model. It is one of the rare sites that allow the user to find dual-objective optimal designs that compromise on 2 objectives according to their relative importance; this is accomplished by assigning a weight (W) between 0 and 1, with a larger weight representing a greater interest in that objective. If the weight is 0 or 1, it reduces to finding a single-objective optimal design. In addition, the reported optimal approximate designs are shown to be optimal using a plot over the dose range that theoretically confirms the optimality of the design. Because the model is nonlinear, nominal values are required for the model parameters. Up to 5 sets of nominal values are allowed, and the user finds a Bayesian optimal design by assigning a prior distribution to the 5 sets of nominal values. If a single set of nominal values is used, we obtain the usual locally optimal designs. Such self-created web links for solving selected types of design problems and conducting specific data analyses for clinical trials are available but not systematically documented in the literature. Another interactive site that uses R-based *shiny* apps is provided [32]. The site is relatively new and is now continuously enhanced with additional features. It is oriented toward toxicology studies but some of the models are also appropriate for clinical trials. The capabilities of the sites include model fitting and finding locally optimal designs for studies with 2 objectives, and one is equally or more important than the other.

Another useful link is available via the *rpact* R package [33] created by Wassmer and Pahlke [34]. It provides a comprehensive tutorial on confirmatory adaptive clinical trial designs, simulations, and analyses for clinical studies. It consists of a validated, open-source, and free-of-charge R software package for clinical trial planning, simulation tools, design evaluation, and data analysis. It can be used to perform sample size and power calculation for both fixed sample designs and designs with interim analysis stages. In addition, it offers simulation tools for means, rates, and survival data to evaluate the adaptive sample size or event number recalculations based on conditional power, treatment selection strategies in multiarm trials, and enrichment designs. Ryeznik et al [35] provided a software package for designing randomized response-adaptive clinical trials with time-to-event outcomes, and codes were downloaded directly from the journal website [35].

Commercial statistical packages also provide built-in commands for searching different types of designs and a myriad of data analytical tools for making inferences in different types of clinical trials. For example, in STATA, there is a single command that finds a phase 1 design using the continual assessment method. A skeleton has to be specified, along with a single parameter toxicity model, where after each response, the single parameter is updated using a Bayesian paradigm. If there are 5 doses at 0.05, 0.1, 0.17, 0.3, and 0.4 to be explored for finding the maximum tolerated dose; the assumed probability of having a toxic response at these levels is 1, 2.5, 5, 10, and 15; and an exponential prior is used for the single parameter in the power model for toxicity using a set of 90 grid points for integration, one simply types “crm y dose, s(0.05 0.1 0.17 0.3 0.4) dose(1 2.5 5 10 15) target(0.3) model(power) inv(power) prior(exponential) pmean(1) quad(90) g” and the simulated results are displayed in tabular and graphical forms.

In summary, interactive websites are useful because they allow users to input design parameters and the design appears quickly after a few strokes on the keyboard. They are especially helpful for biomedical researchers who understand what the designs do and want to find the designs quickly and have little interest in their technical construction. Unlike MD Anderson’s website, they do not require the user to install new programs and read help manuals before the codes are run to find the desired designs. In the era of digital health, such web-based tools can now blunt the old and common criticism that adaptive designs require a statistician to randomize patients or cohorts of patients in the trial since the process can now be safely automated. However, interactive websites generally need to be thoughtfully constructed and can be costly to maintain as technology changes and old programs may become security threats and need to be taken down or totally revamped. This was the experience of one of the coauthors.

### Metaheuristics for Designing Efficient Clinical Studies

#### Overview

Increasingly, metaheuristics is used to tackle all types of optimization problems, particularly in engineering and computer science. Algorithms motivated by nature behavior are called nature-inspired metaheuristic algorithms, and they are especially popular because of the voluminous reports of their flexibility and successes in tackling all kinds of challenging optimization problems. There are many monographs on metaheuristics, such as the studies by Yang [36] and Engelbrecht [37], and how they may be hybridized with other algorithms for a more effective search [38]. Recent review papers on metaheuristic algorithms include the studies by Bonyadi and Michalewicz [39] and Korani and Mouhoub [40]. Metaheuristics with a focused application area is also available. For example, Nakib et al [41] cataloged applications of metaheuristics in a book for medicine and biology, the study by Yang [36] was concerned with engineering applications, Sun et al [42] focused on estimation problems in systems biology, and Mendes et al [43] focused on agricultural problems. The meteoric rise of metaheuristics is well documented in the studies by Whitacre [44,45].

Exemplary nature-inspired algorithms are PSO proposed by Kennedy and Eberhart [46] and differential evolutionary proposed by Storn and Price [47]. The first is swarm based and simulates how a flock of birds searches for food on the ground, and the other is evolutionary, based on the evolution of genetics. These algorithms, as the names suggest, are motivated by nature and are intriguing because they tend to, with repeated runs, be able to find the global optimum or close to it, although they rarely have rigorous mathematical proofs of convergence to the global optimum. All nature-inspired metaheuristic algorithms are literally free of assumptions; they have tuning parameters and stochastic elements in the 2 or 3 relatively simple equations that define how the algorithm works to simulate the movement of animals. Increasingly, there are calls to apply metaheuristics and machine learning tools for pharmaceutical research and they include past studies [48-50].

Nature-inspired metaheuristic algorithms are not without problems, and they include how to skillfully choose the values of the tuning parameters and how to choose the type of stochastic components in them. In addition, search candidates can move outside the search domain and must be returned to the domain judiciously. This issue can affect the performance of PSO, particularly when solving high-dimensional optimal design problems. Unlike the first 2 problems concerning the choice of values for the tuning parameters and the choice of stochastic components, where some progress has been made, the third issue of how to bring back out-of-bounds candidate solutions to the search domain has not been investigated. After we briefly review some recent uses of metaheuristics in clinical trials, the following subsection investigates this important issue when PSO is used to find various types of optimal designs for nonlinear models with 3 to 4 parameters useful for biomedical studies.

We have cited some recent applications of PSO to identify more flexible or efficient designs for clinical trials. Lange and Schmidli [51] and Qiu et al [52] were among the first to use optimal design theories and PSO to construct efficient designs. The first set of authors focused on applications for biological clinical trials, and the latter set of authors provided several applications for finding optimal designs for a variety of models useful for biomedical studies. For more complicated design problems, Chen et al [53] demonstrated how PSO can be modified or hybridized with another algorithm to search for an optimal design that best discriminates among nonlinear models under various error distributional assumptions with applications in toxicology. In all cases, the abovementioned studies focused on optimal approximate designs, which are large-sample optimal designs, and view designs as probability measures in a design space. An advantage of working with such designs is that when the design criterion is convex as a function of the information matrix of the design, we have a convex optimization problem, and the optimality of an approximate design can be readily verified [54]. An overview of how to use PSO to solve various design problems in statistics is present in the study by Chen et al [55].

To elaborate, we provide 2 recent applications of PSO to identify more flexible and efficient designs for medical problems. The first application used PSO to provide more flexibility to the celebrated Simon Two-Stage Design for a phase 2 trial [27], and the second application used PSO to solve a challenging design problem to estimate the most efficacious dose (MED) for the continuation ratio (CR) model.

At stage 1 of the original Simon design, the drug is tested to determine whether there is potential usefulness of the drug. If there are too few responders, the drug is deemed ineffective and the trial is terminated. If there are enough responders in stage 1, the trial moves on to the second stage, where additional patients will be recruited and tested. If the cumulative percentage of responders at the end of stage 2 is large enough, the drug will progress to phase 3 for large-scale testing; otherwise, the drug is deemed ineffective and the trial is terminated at stage 2. The null hypothesis at stage 1 is to test whether the response rate is equal to a user-specified value of p_0_, and at the second stage, we used the cumulative data and tested a second null hypothesis—whether the response rate is equal to p_1_ where p_1_>p_0_. The statistical questions to be answered are the given type 1 and 2 errors for testing the 2 hypotheses, what is the sample size (*n*_1_ for stage 1) and how many responders (*r*_1_) are needed in stage 1 to advance to the second stage? There are also questions about the number of additional participants (*n* − *n*_1_) to recruit for stage 2 and whether additional responders (*r* − *r*_1_) are needed in stage 2 to reject the null hypothesis.

The optimization problem is to find the optimal integer values of *n*_1_*, n, r*_1_, and *n* that meet the 2 sets of error constraints, which can be solved by a greedy search using binomial probabilities. Subsequent criticism concerns how p_1_ is selected in practice. To overcome this difficulty, Lin and Shih [56] proposed 2 null values, p_10_ and p_20_, for testing in stage 2, and only one of them will be tested, depending on the stage 1 results. There are now 6 integer values to optimize subject to 6 error constraints. The authors noted that their greedy search approach exhausted all the computing resources at that time. To further mitigate the uncertainty of specifying the value of p_1_ in the second stage, Kim and Wong [57] proposed specifying in advance 3 possible values of p_1_, and only one of the null hypotheses will be tested, depending on the stage 1 result. Using a hybrid version of PSO, they were able to verify that their results were the same as earlier results when there were only 1 or 2 null hypotheses, and their results satisfied the 4 sets of prespecified type 1 and 2 error rates, which can vary depending on the set of hypotheses to be tested; details are available in the study by Kim and Wong [57].

Increasingly, phases 1 and 2 are comingled to assess toxicity and efficacy to save cost in a clinical trial. Many models have been proposed to incorporate toxicity and efficacy concerns at the onset of the study. Some have a model for modeling toxicity and another for modeling efficacy and are then linked using joint and conditional probabilities. An early work using this approach is the study by Heise and Myers [58] and a recent approach is the study by Aout and Seroutou [59]. More recently, Fedorov et al [60] tackled a more challenging problem when they sought a model-based optimal design to incorporate a continuous (efficacy) and a discrete response (toxicity), and the 2 responses are correlated.

The CR model is a dose-response model that allows for a 3-category outcome as the dose levels vary. Fan and Chaloner [61] discussed the optimal designs for estimating model parameters or a selected function of the model parameters for the CR model on an unbounded dose interval. Qui and Wong [62] modified the PSO and applied it to find ≥2 objectives when some are more important than others on any user-specified interval for the CR model. Specifically, PSO was used to find locally optimal designs for estimating the 4 parameters in the CR model, MED, and the maximum tolerated dose. The latter 2 objectives are convex functions of the information matrices, so equivalence theorems can be used to confirm the optimality of the design.

#### Repair Mechanisms Comparison in PSO

There are 2 types of repair mechanisms commonly used in the literature: random repair and boundary repair [63]. Let us assume that PSO searches a *J*-dimensional dose space



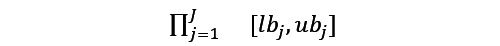



where *ub_j_* and lb*_j_* are the upper and lower bounds of the *j*th dimension, respectively.

The *i*th particle’s position at time *t* is a vector



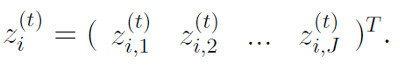



The random repair, as the name indicates, randomly assigns a position within the dose space when a particle wanders outside of the design space,



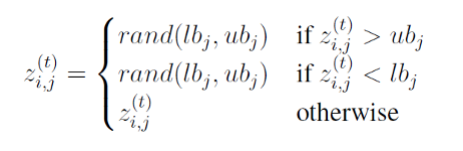



where *z*^(^*^t^*^)^ is the *j*th component of the *i*th particle’s position at time *t*, and *rand*(*lb_j_, ub_j_*) is a random variable from a uniform distribution on [*lb_j_, ub_j_*]. This repair mechanism has 2 direct effects on the swarm movements: (1) increasing the *i*th particle’s velocity *v*^(^*^t^*^)^ at time *t* and (2) increasing |*p_i,j_* − *z*^(^*^t^*^)^| and |*p_g,j_* − *z*^(^*^t^*^)^|. Both effects increase the energy of the swarm and “disturb the swarm into chaos state,” thereby slow down the convergence speed to the global optimum [63].

Another type of repair strategy in PSO is boundary repair. This strategy pulls errant particles back to the nearest boundary of the design space:



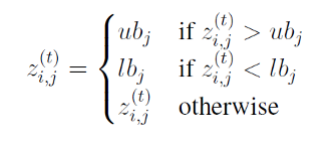



The effects of boundary repair on swarm movements are opposite to what random repair does: (1) decreasing the velocity *v*^(^*^t^*^)^ and (2) decreasing |*p_i,j_* − *z*^(^*^t^*^)^| and |*p_g,j_* − *z*^(^*^t^*^)^|. Both effects directly decrease the energy of the swarm. The boundary of the dose space acts as a quasi-gravity center that attracts particles current and the following positions until the population’s best position is no longer at the boundary. Such a repair mechanism “accelerates the swarm into an equilibrium state and may lead to premature convergence” [63]. It is known that locally *D* and *c*-optimal designs frequently have support points at or near the boundary of the design space [64]. This suggests that bringing back out-of-bounds particles to a random point in the boundary may be a good strategy. We call this modification the boundary repair mechanism.

To compare the performances of the 2 repair mechanisms, we conducted simulation studies to investigate their choice of locally finding *D*- and *c*-optimal designs for various statistical models. This study was, in part, first motivated by their impact on the search for a *D*-optimal design for a linear model with 2 variables, given in the following equation:

Ey = *β*_0_ + *β*_1_*x*_1_ + *β*_2_*x*_2_ + *β*_11_*x*_1_^2^ + *β*_12_*x*_1_*x*_2_*, x* ∈ *χ* = [−1*,* 1] × [0*,* 1]*.*

**Figure 1:**
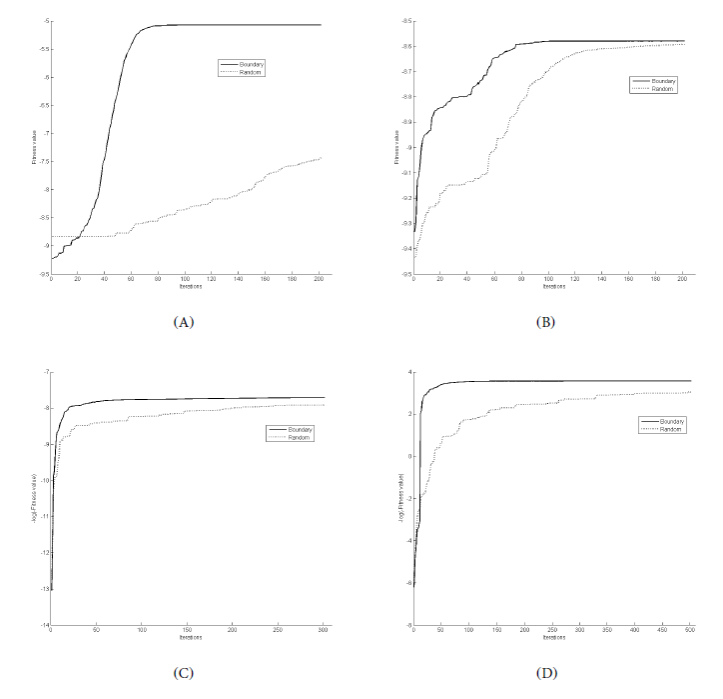
Convergence rates of the two repair mechanisms in PSO for finding (A) the D-optimal design for the 2-variable linear model on χ=[-1,1] X [0,1] and (B) the locally D-optimal designs for the logistic quadratic model with nominal parameters α=3, β = 5, and µ=0 on χ=[-1,1]; (C) locally area under the curve optimal design for the compartmental model with nominal parameters θ_1_=0.05884, θ_2_=4.298; and θ_3_=21.8; and (D) the local t_max_ optimal design for the compartmental model in (C).

Figure 1A plots the fitness values of the *D*-optimality design criterion versus the iteration numbers. For this linear model with 2 interactive variables, we observed that the strategy of randomly assigning out-of-boundary particles into the design space clearly underperforms when compared to the other strategy of pulling back articles to the boundary of the design space. This finding is not surprising because *D*-optimal designs for linear models invariably have design points at the boundary [64]. This led us to investigate whether such underperformance by one strategy applies to finding optimal designs for commonly used nonlinear models and whether such an observation is confined to *D*-optimality only.

We applied PSO to find various types of optimal designs for 3-parameter and 4-parameter nonlinear models. We locally found *D*-optimal designs for estimating all parameters in the model, and we constructed *c*-optimal designs for estimating (1) the average time a drug stays in the targeted compartment; (2) the time to reach its maximum concentration in a 3-parameter compartmental model; and (3) the MED for a continuation model that incorporates toxicity and efficacy probabilities in the model as the drug concentration, on the log scale, varies. The MED sought is the dose that produces the most efficacy at a user-specified level of toxicity deemed acceptable. This design problem is complicated because an analytical form of the estimated MED is not available, and we have to resort to the implicit function theorem to find the gradient of the MED function, which is needed to find the local *c*-optimal design [61]. In either case, we have a statistical model with one variable *x* representing time or concentration dose levels, and we compared the 2 pulling-back strategies in PSO for finding an efficient design under the *D* or *c*-optimality criterion. These models of various complexities are as follows:

1. Compartmental model [54]
  Ey = *θ*_3_(exp(−*θ*_2_*x*) − exp(−*θ*_1_*x*))*, x* ∈ *χ* = [0*,* 20] **(1)**with nominal values of *θ*_1_=0.05884, *θ*_2_=4.298, and *θ*_3_=21.8.
2. Logistic quadratic model

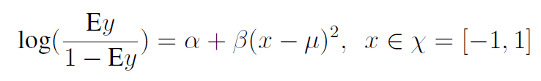

**(2)**
  with nominal values of *α*=3*,* β=−5, and µ=0.
3. Heteroscedastic 4-parameter Hill model [65]

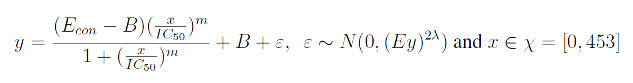

**(3)**
  with nominal values of E*_con_*=1.7*,* B=0.137*,* IC_50_=0.453*,* m=−0.825 and λ=3.
4. CR model [61]
  log(π_3_(x) */* (1 − π_3_(x))) = a_1_ + b_1_x **(4)**
  log(π_2_(x) */* π_1_(x)) = a_2_ + b_2_x, x∈χ = [−10*,* 10] with nominal values a_1_=0, b_1_=b_2_=1, a_2_=5 for the constant slope case, and nominal values a_1_=−3.3, b_1_=0.5, a_2_=3.8, and b_2_=1 for the non–constant slopes case.

We applied PSO to find the MED when the CR model in equation 4 has a nonconstant slope and noted that the MED sought is the solution to the equation *b*_2_(1 + exp(−*a*_1_ − *b*_1_*x*)) − *b*_1_(1 + exp(*a*_2_ + *b*_2_*x*)) = 0, which does not have a closed form. We also used PSO to find *c*-optimal designs for estimating 2 functions *c*(*θ*) of the model parameters *θ*. The 2 functions of interest are the average time the drug spends in the target compartment and the time required to reach the maximum concentration in the target compartment. These 2 functions are determined by integrating the mean response function from 0 to +∞ to obtain the area under the curve and using calculus to obtain the time (*t_max_*) required to attain the maximum mean response function of the compartmental model in (1). A direct calculation shows the following:



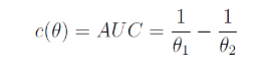



or



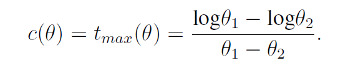



**Figure 2:**
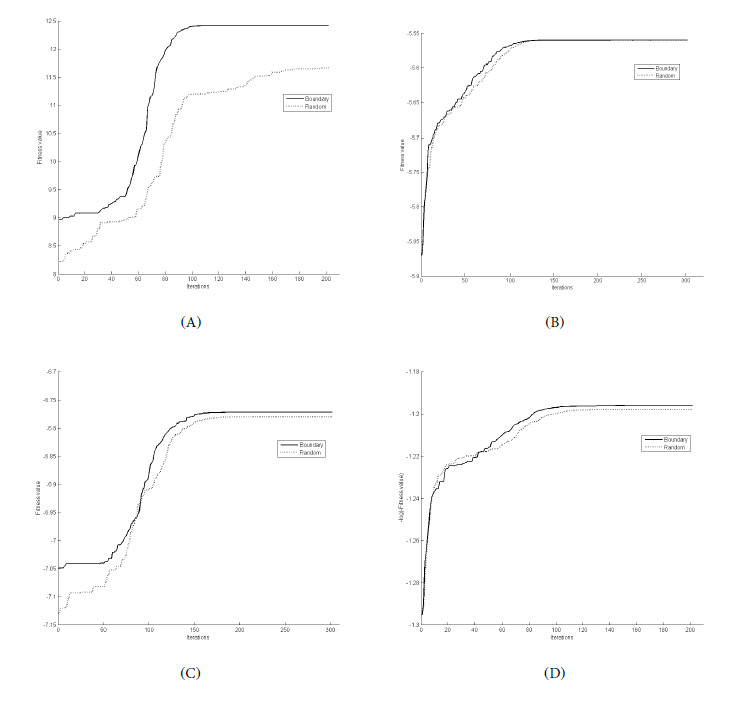
Convergence rates of the two repair mechanisms in PSO for finding D- and c-optimal designs for various 4-parameter nonlinear models: (A) locally D-optimal design for the Hill model with E_con_=1.7, b=0.137, IC_50_=0.453, m=0.825, λ=3 on χ=[0, 453]; (B) locally D-optimal design for the continuation ratio (CR) model on χ=[-10, 10] with a constant slope and nominal parameters a_1_=0, b_1_=1, a_2_=5, b_2_=1; (C) locally D-optimal design for the CR model on χ=[-10, 10] with unequal slopes with nominal parameters a_1_=3.3, b_1_=0.5, a_2_=3.8, b_2_=1; and (D) locally c-optimal design for estimating the most efficacious dose for the CR model in (C).

The fitness values of the PSO-generated designs under the local D and *c*-optimality criteria are, respectively, log(*I*(*ξ, θ*)) and −log(−∇*^T^ c*(θ)*I*^−1^(*ξ, θ*)∇*c*(θ)). We ran PSO 20 times with the random or boundary repair mechanism to find the locally *D*- and *c*-optimal designs for the above models. In each replicate, the best fitness values found by the entire flock at every iteration were recorded. Figures 1 and 2 show the average best fitness values over 20 replicates versus the number of iterations for the 2 repair mechanisms for the different models using the *D* or *c*-optimality criteria. The solid curves are generated by PSO with the boundary repair mechanism, and the dotted curves are generated by PSO with the random repair mechanism.

Figures 1B and 2A show that PSO with boundary repair consistently converges faster than PSO with a random repair under the *D*-optimality criterion for the univariable models. The linear model with 2 regressors exhibits a greater difference in performance when different pull-back strategies are used in the PSO (Figure 1A). For complicated models, our experience is that PSO with a random repair is unable to find the optimal design regardless of the maximum number of iterations and flock size we chose. For the CR model, where we want to find locally *D*-optimal designs for constant (Figure 2B) and nonconstant slope models (Figure 2C) and a c-optimal design for estimating MED (Figure 2D), we observed that although PSO with boundary repair still outperforms the other strategy, the difference in their performance seems to be increasingly smaller as PSO progresses.

### Conclusions

We conclude with a summary of the benefits of adaptive designs and metaheuristics and offer commentaries on their use in practice.

Adaptive clinical trial designs provide numerous advantages over traditional fixed designs, including increased efficiency, enhanced ethical considerations, improved patient safety, increased flexibility, increased probability of success in identifying effective treatments, and the ability to address multiple research questions. Specifically, adaptive designs can increase the statistical power of a trial, reduce the number of patients required, decrease trial duration, and lower overall costs. Furthermore, adaptive designs can increase the likelihood of patients receiving superior treatment and decrease the risk of exposing them to unsafe or ineffective treatments. In addition, adaptive designs allow for flexibility in adjusting the sample size, randomization ratios, and end point analyses based on the interim data analysis. The ability to adapt the design of a trial also enables researchers to address multiple research questions within the same trial by testing multiple hypotheses and can increase the probability of identifying effective treatments. When reporting results from adaptive clinical trials, it should be clarified whether the adaptation was planned or unplanned, what was the rationale behind the adaptation, when the adaptation was made, and whether it was applied to some or all data. Information regarding whether the data were blinded should also be provided. One also needs to describe who made the decision regarding adaptation, deviations from the planned process, and consistency of results before versus after the adaptation. Finally, one needs to discuss the potential biases induced by the adaptation, strategies to avoid operational bias, and the effects on error control and multiplicity context.

To elaborate on the above remarks, consider RAR trials that are, by construction, more ethical than traditional designs. Compared with traditional designs, they are generally more cost and time efficient. Another merit is that they can potentially improve trial recruitment because more patients can be assigned to better treatment. RAR trials also provide a fruitful area for further research, and there are already some initial extensions of RAR trials for multiarmed survival trials [35,66,67] or for trials with a couple of objectives with competing interests [68]. It is also worth noting that Tehranisa and Meurer [23] found that clinical trials that implement an RAR design will attract significantly higher participation than standard randomization, which might be particularly beneficial to rare disease clinical trials. In summary, adaptive designs are flexible designs that offer many benefits over traditional designs and should be more widely implemented in practice.

Metaheuristics is already widely used across disciplines, including in artificial intelligence research, and its use in finding efficient studies is just beginning. Metaheuristic algorithms are general purpose optimization algorithms with minimal or no assumptions required, and they have been shown in the engineering and computer science literature that they work well in tackling complex optimization problems, including high-dimensional problems that naturally occur in the internet era. A common criticism is that there are many such algorithms and almost all work similarly by exploring the search domain for the optimum and exploiting the sites near a global optimum in different ways. Because they are differently motivated, some tend to be better in solving certain types of optimization problems. Unfortunately, there is rarely a clear-cut answer, so researchers should be familiar with many types of metaheuristic algorithms, including mathematical programming tools, such as semidefinite programming methods, and know the strengths and weaknesses of each algorithm. Hybridization is a technique to enhance the performance of a metaheuristic algorithm by combining its best features with one or more other types of algorithms that can compensate for some of its weaknesses. Blum and Raidl [38] provide details and real examples.

There is no proof of convergence for almost all metaheuristic algorithms, and there is no clear way of confirming the optimality of a solution. Consequently, it is important to verify the optimum found from a metaheuristic algorithm, particularly when there is no theoretical tool to do so. At a minimum, we recommend 2 checks. The first is to modify the algorithm by changing its tuning parameters, flock size, the number of iterations, and particular strategies of the algorithm to ascertain whether the same or similar optimum is generated. For instance, we demonstrated that different strategies for bringing back out-of-domain particles can affect the quality of the found optimum; in particular, Figure 1A shows that interestingly the most marked difference in the 2 strategies was observed for the linear model with 2 interacting variables. The second is to run different metaheuristic algorithms and observe whether they provide similar answers. In sum, they are intriguing algorithms, and their use should be more widespread in clinical trial research settings.

## Abbreviations

ASD: adaptive seamless phase 2/3 trial design
CR: continuation ratio
DBCD: doubly adaptive biased coin design
DTR: dynamic treatment regime
FDA: Food and Drug Administration
INHANCE: Indacaterol to Help Achieve New Chronic Obstructive Pulmonary Disease Treatment Excellence
MED: most efficacious dose
PSO: particle swarm optimization
RAR: response-adaptive randomization
SMART: Sequential Multiple Assignment Randomized Trial
